# From Glucose Transport to Microbial Modulation: The Impact of Sodium Glucose Co-Transporter-2 Inhibitors on the Gut Microbiota

**DOI:** 10.3390/medsci14010022

**Published:** 2026-01-03

**Authors:** Mina Y. George, Nada K. Gamal, Kerolos Safwat, Mohamed Mamdouh, Ahmed AbdElFatah, Abdelrahman Atallah, Claudio Cerchione

**Affiliations:** 1 Department of Pharmacology and Toxicology, Faculty of Pharmacy, Ain Shams University, Cairo 11566, Egypt; 2 PharmD Clinical Program, Faculty of Pharmacy, Ain Shams University, Cairo 11566, Egypt; 3 Hematology Unit, Istituto Romagnolo per lo Studio dei Tumori “Dino Amadori”—IRST IRCCS, Via Piero Maroncelli 40, 47014 Meldola, FC, Italy

**Keywords:** SGLT-2 inhibitors, microbiome, gut, dysbiosis, microbial composition

## Abstract

**Background:** Sodium glucose co-transporter-2 (SGLT-2) inhibitors are antihyperglycemic drugs used in type 2 diabetes mellitus management, and they have associated cardiovascular and renal advantages beyond their glucose-lowering effects, with maintained proof linking gut microbiota modulation to their multiple therapeutic benefits. **Aim:** This review aims to deliver an overview of the current knowledge regarding the relationship between SGLT-2 inhibitors and the gut microbiota and how this interplay impacts the gut–organ axes such as the lung, heart, brain, liver, and hematological system. **Methodology:** A literature review was performed in Web of Science, PubMed, and Google Scholar to discover studies that assessed the effects of SGLT-2 inhibitors on gut microbiota composition, microbial metabolites, and associated systemic consequences. Results: SGLT-2 inhibitors modulate gut microbiota and its driven metabolites, strengthening the barrier integrity and alleviating endotoxemia, inflammation, and oxidative stress, resulting in beneficial outcomes across the different gut–organ axes. **Conclusion:** Gut microbiota modulation is an emerging approach in mediating the multifaceted beneficial impacts of SGLT-2 inhibitors, revealing that their effectiveness goes beyond glycemic control. Future research should concentrate on the microbial taxa and metabolites that mediate these impacts and testing combination approaches that target SGLT-2 pathways and gut microbiota to enhance preservation of different organs.

## 1. Introduction

Sodium glucose transporters (SGLTs) are proteins responsible for the regulation of glucose reabsorption in the human body. SGLT subtypes are found in various regions of the body [1]. SGLT-1 is mainly located on the luminal side of the small intestine, which plays an essential role in glucose transport into the enterocyte, while SGLT-2 is primarily located in the kidney tubules [2].

SGLT-2 inhibitors have been extensively used as antidiabetic drugs. Although their main mechanism involves glucose reabsorption in the kidneys, they possess different degrees of SGLT-1 inhibitory activity [3]. For example, canagliflozin partially inhibits intestinal SGLT-1, causing a reduction in glucose absorption in the small intestine. This results in an increase in glucose transport to the ileum and colon, which in turn affects glucose fermentation in the colon. Glucose fermentation in the colon alters the microbial metabolite balance by enhancing short-chain fatty acids (SCFAs), as well as reducing harmful uremic toxins, such as P-cresyl sulfate and indoxyl sulfate [4]. Additionally, SGLT-2 inhibitors are becoming more widely acknowledged for their role in heart failure treatment via pleiotropic processes apart from glucose reduction. They considerably lower hospitalization and cardiovascular death in patients with both intact and reduced ejection fraction, regardless of diabetes status. Beyond their established cardiometabolic attributes SGLT-2 inhibitors have been reported for their wide variety of pharmacological activities in vivo and in vitro, such as antioxidant, anti-inflammatory, anticancer, cardioprotective, and neuroprotective effects [5,6,7,8].

The gut microbiota influences different physiological and pathological processes in human organs, including the brain, heart, lungs, and kidneys, through complex bidirectional communication networks often referred to as “axes” [9]. Therefore, different axes have been identified in the literature, including gut–lung, gut–kidney, gut–heart, and gut–brain axes. Additionally, SGLT inhibitors were found to improve intestinal permeability by the restoration of tight junction proteins, which reduces the translocation of endotoxin and lipopolysaccharides into the systemic circulation. These effects may explain their cardiovascular and kidney benefits [10].

Regardless of the recognized cardiometabolic effects of SGLT-2 inhibitors, the contribution of gut microbiota regulation in their systemic and diverse therapeutic effects remains insufficiently explored. Contemplating the role of the gut microbiome in modulating inflammation, metabolism, and gut–organ communications, exploring how SGLT-2 inhibitors influence gut-mediated pathways may offer mechanistic insight into their multifaceted effects and help in the identification of novel therapeutic targets across multiple gut–organ axes. So, the aim of the current review is to shed light on different bidirectional connections between the gut and other organs through the microbiome. Also, this review highlights the interplay between SGLT-2 inhibitors and the gut microbiome, as well as explains the possible underlying mechanisms. Additionally, the beneficial therapeutic outcomes following this interplay are also summarized.

## 2. Methodology

The current literature review was written following an online search using Web of Science, NCBI, PubMed, and Google Scholar to locate publications that investigated the interaction between SGLT-2 inhibitors and the gut microbiota. We targeted key terms like SGLT-2 inhibitors, gut microbiota, and microbial metabolites and related topics such as inflammation, gut–lung axis, gut–heart axis, gut–brain axis, and hematopoiesis. The search included 25 research articles, which are English-language primary articles with evidence-based methodologies. Our approach aimed at gathering diverse studies, forming a solid foundation for our review.

## 3. Gut Microbiota and Metabolites

The human gut microbiota is a dynamic and diverse population of commensal and symbiotic microorganisms, exceeding 10^12^ cells per gram in the large intestine. A healthy gut microbiome is important for preserving gut health and maintaining appropriate systemic biological homeostasis since it plays a crucial role in digestion, absorption of nutrients, the integrity of the intestinal barrier, metabolism, and immune system regulation [11]. The majority of the microbiota in the adult human gut is made up of *Bacteroidetes* (e.g., *Bacteroides*) and *Firmicutes* (e.g., *Clostridium*, *Lactobacillus*, *Enterococcus*), with smaller proportions of *Actinobacteria* (e.g., *Bifidobacterium*), *Proteobacteria* (e.g., *Escherichia coli*), *Fusobacteria*, and *Cyanobacteria* [12]. Beneficial commensal bacteria, such as *Faecalibacterium prausnitzii*, *Bifidobacterium* spp., *Roseburia* spp., and *Akkermansia muciniphila*, play a crucial role in maintaining intestinal barrier integrity, regulating immunological homeostasis, and producing anti-inflammatory metabolites [13].

On the other hand, when disproportionately enriched, pathogenic microbes such as *Escherichia coli*, *Clostridium difficile*, *Streptococcus* species, and *Enterococcus* species can cause tissue damage and inflammation [14]. Any departure from the typical composition of gut microbiota, known as “microbial dysbiosis,” is characterized by an imbalance or decline in the relative abundance of *Firmicutes* and *Bacteroidetes* [15].

Numerous conditions, such as metabolic, inflammatory, cardiovascular, hepatic, renal, hematological, and neurological problems, have been linked to dysbiosis [16]. By compromising the integrity of the intestinal barrier, this disruption makes it easier for microbial products like lipopolysaccharide (LPS) to translocate throughout the body, activating Toll-like receptor 4 (TLR4) signaling [17,18,19,20,21] and causing chronic inflammation [22]. Additionally, the microbial metabolites mediate several microbial-driven activities, especially the short-chain fatty acids (SCFAs), including butyrate, propionate, and acetate. SCFAs preserve the intestinal barrier, manage energy metabolism, and modulate both innate and adaptive immune responses [23].

## 4. Bidirectional Connection of the Gut

**Figure 1:**
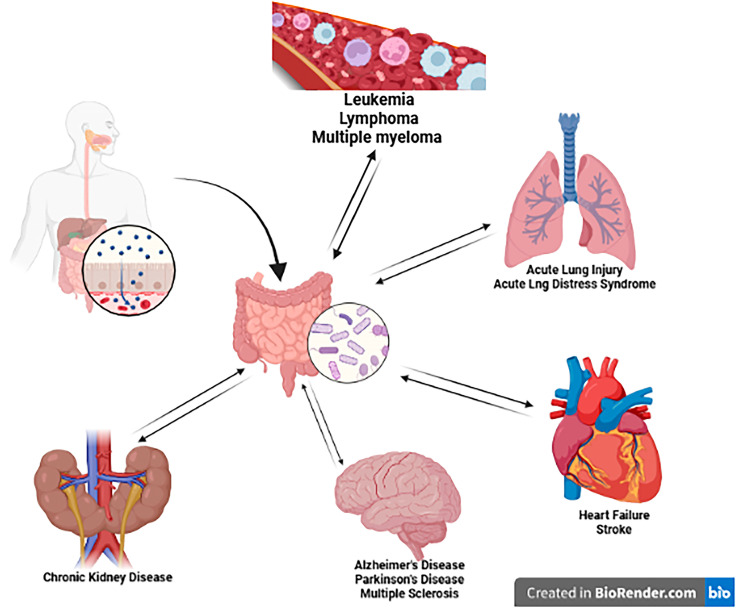
The bidirectional communication between the gut and other body organs through microbiome modulation. Created in https://BioRender.com.

Through the microbiome, the gut can form bidirectional connections with other organs, known as the axis. The gut was found to form bidirectional connections with the lungs, the kidneys, the heart, the brain, and the blood (Figure 1).

### 4.1. Gut–Lung Axis

The gut–lung axis represents the connection between the gastrointestinal tract and the respiratory system. This connection is mainly mediated by microbiota metabolites and the body’s immune system [24]. When the intestinal barrier is compromised due to multiple factors, gut bacteria and endotoxins can migrate to the lungs through the bloodstream and lymphatic systems, triggering a proinflammatory response that damages lung tissue [25]. This may result in serious conditions such as acute lung injury (ALI) and acute respiratory distress syndrome (ARDS), acute inflammatory lung disorders triggered by several factors, including sepsis, trauma, or shock. Both conditions are characterized by hypoxemia and inflammation, leading to difficulty breathing and impairing oxygen exchange, which makes them life-threatening conditions if untreated [26]. The gut microbiome and its metabolites play a critical role in ALI/ARDS onset and progression. A recent study showed that there is an imbalance between beneficial and pathogenic bacteria in ARDS patients. Beneficial commensal bacteria, such as *Faecalibacterium prausnitzii* and *Bifidobacterium*, tend to decrease, while pathogenic or conditionally pathogenic bacteria, including *Escherichia coli* and *Streptococcus*, are found to be increased. As a result, an alteration in SCFAs occurs, which modulates the gut–lung axis by hampering inflammation and oxidative stress and maintaining the intestinal barrier integrity and permeability. By activating G-protein-coupled receptors (GPR41, GPR43, and GPR109A) on immune cells, SCFAs reduce proinflammatory cytokines like TNF-α, IL-6, and IL-1β and decrease neutrophil recruitment systemically. Moreover, SCFAs improve regulatory T-cell differentiation, reduce oxidative stress and neutrophil extracellular trap formation, maintain the integrity of the alveolar–capillary barrier, and modulate alveolar macrophage polarization toward an anti-inflammatory M2 phenotype within the lung, all of which contribute to the progression of ALI/ARDS [26].

### 4.2. Gut–Kidney Axis

The gut microbiota can influence the kidneys and has a relation to kidney-related diseases, especially in chronic kidney disease (CKD), through what is known as the gut–kidney axis. When this axis becomes disrupted due to dysbiosis, an imbalance in the gut microbiota, combined with impaired gut permeability, leads to an increase in endotoxin, oxidative stress, and lipopolysaccharide (LPS) in the systemic circulation. This could stimulate the immune system and enhance the production of proinflammatory cytokines, such as interleukin (IL)-6 and tumor necrosis factor-α (TNF-α), inducing systemic inflammation [27].

Some therapies that target the gut–kidney axis focus on restoring the equilibrium of microbiota, reducing the level of uremic toxins, and improving gut permeability. Probiotics and prebiotics presented promising results in enhancing beneficial bacteria and hindering harmful microbial metabolites. Moreover, a low-protein, high-fiber, and plant-based diet showed an elevation in SCFAs, which leads to reduced inflammation and oxidative stress. Other treatments for constipation and fecal microbiota transplantation (FMT) are being tested for their ability to correct microbial imbalances and decrease toxin buildup in CKD patients. Although these treatments are encouraging, more research is needed to fully understand how specific microbes affect kidney function and health [27].

### 4.3. Gut–Heart Axis

There is a growing interest in the connection between gut health and heart function. Jaimez-Alvarado et al. found that patients with gastrointestinal tract diseases, such as gastroesophageal reflux disease and inflammatory bowel disease, have a higher risk of developing cardiovascular diseases like heart failure and stroke [28]. A recent animal study showed that there is a decrease in the *Firmicutes*/*Bacteroidetes* ratio in heart-failure-induced mice that have been treated with dapagliflozin, an SGLT-2 inhibitor, resulting in reduced inflammation and cardiac fibrosis in mice. These findings suggest that dapagliflozin may modulate gut microbiota, contributing to heart failure treatment [28].

### 4.4. Gut–Brain Axis

The gut–brain axis is a bidirectional communication pathway between the gut microbiome and the central nervous system. This system is managed by many immune, hormonal, and neuronal connections, including immune cells, the vagus nerve, the neuroendocrine system, and gut bacterial metabolites/products [29]. During dysbiosis, the pathways of the gut–brain axis become dysregulated, leading to altered permeability of the blood–brain barrier and increased susceptibility to immunological and neurological diseases, such as Alzheimer’s disease, Parkinson’s disease, and multiple sclerosis. These diseases are characterized by activation of the immune system due to exposure to microbiota metabolites, leading to the production of proinflammatory cytokines like IL-1β and IL-18 [30].

### 4.5. Gut–Hematopoietic Axis

Studies have shown that there is a link between the gut microbiota and the hematopoietic system, which addresses the effect of gut microbiota on the activity of hematopoietic stem and progenitor cells (HSPCs), immune cell development, and maintaining blood homeostasis through their metabolites and structural features [31]. Gut microbiota alterations were seen in subjects suffering from hematological malignancies, which affected onset and progression [32]. In leukemia, microbial diversity is altered. An enrichment of *Bacteroides* species and decreased levels of helpful *Fusobacterium naviforme* and *Roseburia faecis* in children were found [33]. This dysbiosis was linked to the exacerbation of leukemia since it disrupted the intestinal barrier, allowing LPS translocation and resulting in systemic inflammation and progression of leukemia. However, restoration of the compromised barrier using butyrate was shown to lower circulating LPS and suppress leukemic progression [34]. Similarly, patients with lymphoma showed dysbiosis. Individuals with B-cell non-Hodgkin lymphoma demonstrated a higher abundance of *Bacteroidetes* and lower *Firmicutes*. Moreover, the diversity was notably lower in aggressive forms of B-cell lymphoma [35]. Interestingly, Shi et al. revealed that the equilibrium between two bacteria in the gut, *Streptococcus parasanguinis* and *Romboutsia timonensis,* can be employed as a diagnostic marker for natural killer/T-cell lymphoma by establishing the *Streptococcus parasanguinis*–*Romboutsia timonensis* index (SRI). Patients who had increased SRI values had shorter progression-free survival [36].

Multiple myeloma (MM) has also shown microbial alterations. Studies have indicated that gut dysbiosis plays a role in MM development and progression. MM patients possess reduced numbers of beneficial butyrate-producing bacteria, including *Clostridium butyricum* and *Anaerostipes hadrus*, enhanced levels of *Streptococcus*, *Klebsiella*, and *Pseudomonas aeruginosa*, and *Actinobacteria* and *Proteobacteria* in lower numbers. These alterations in microbiota restrict SCFA production, which helps to maintain immunity and manage inflammation, creating a bone marrow milieu conducive to the formation of malignant plasma cells [37].

## 5. Interplay Between SGLT-2 Inhibitors and Gut Microbiome

Several studies have highlighted the modulatory role of SGLT-2 inhibitors in the gut microbiome in preclinical research. The effects of SGLT-2 inhibitors on gut microbiome composition are summarized below (Table 1).

### 5.1. Dapagliflozin

In heart-failure-affected mice, there was a marked change in the dysbiotic ratio of *Firmicutes* to *Bacteroidetes*, along with decreased Shannon and Chao1 indices, which indicate lower diversity, as well as increased Simpson index depicting domination by a select few microbes. Dapagliflozin treatment reversed the dysbiosis of heart failure by correcting the *Firmicutes* to *Bacteroidetes* ratio and diversity indices. PCoA and NMDS β-diversity analyses indicated clustering of microbial groups, suggesting modification of the gut ecosystem by dapagliflozin. There was heart-failure-associated augmentation in the genera *Rikenella* and *Mucispirillum*, which are of potential inflammatory and metabolic significance. On the other side, dapagliflozin treatment enhanced *Desulfovibrio*, AF12, and *Paraprevotella*. *Desulfovibrio* and *Paraprevotella* are usually recruited to the gut surface and associated with sulfur metabolism and inflammation, respectively, while the noted relationships between *Paraprevotella* and AF12—possibly protective—imply that there were some complexities to the microbiota changes such that they represent predominantly beneficial changes, while some compensatory turbulence was signaled during the development of heart failure [38].

The functional KEGG and PICRUSt analyses support that the altered microbiota found in heart-failure-induced and dapagliflozin-treated mice engaged in various functional metabolic signaling pathways relevant to fermentation, amino acid biosynthesis, carbohydrate degradation, lipid biosynthesis, and vitamin metabolism, like biotin biosynthesis. Interestingly, several microbial pathways—biotin biosynthesis II, taurine degradation, and sulfolactate metabolism—were found to be elevated following dapagliflozin treatment, as would be expected with overall upsurge of microbial sulfur metabolism, and became analogous with increased microbial sulfur metabolism and nutrient processing.

The differences observed in microbial pathways could potentially lead to systemic inflammation and affect metabolic health. In further evidence for a causal role for the microbiota, FMT from heart-failure-affected mice to germ-free mice duplicated parameters of cardiovascular dysfunction, which also were decreased when the FMT donor was treated with dapagliflozin. As such, there appears to be a direct effect of microbiota on the modulation of an altered cardiac outcome. Overall, the data suggests that some benefits of dapagliflozin, in addition to glucose control, would involve a complex reorganization of the gut microbiome. Certain genera, including AF12, and functional pathways related to vitamin and sulfur metabolism may be viable therapeutic targets and/or biomarkers for heart failure, although bacteria such as *Desulfovibrio* and *Paraprevotella* may warrant further caution due to their potentially competing roles. It must be noted, however, that as this study was conducted in mice, further clinical work in humans is necessary to validate these findings [38].

In another study, the effect of the antidiabetic agent dapagliflozin on gut flora in type 2 diabetic rats was observed. The researchers collected fecal samples and, via 16S rRNA gene sequencing, studied the microbial community of the gut. Despite that both dapagliflozin and metformin reduced blood sugar levels, their impacts on the gut microbial communities were diverse. Unlike metformin, dapagliflozin did not promote the growth of the most commonly beneficial fecal bacteria, such as *Bifidobacterium* or *Lactobacillus*, but it did, however, slightly increased microbial diversity, as observed using the Shannon index. Significantly, however, dapagliflozin increased *Proteobacteria*, especially *Desulfovibrionaceae*, since they usually show an inflammatory trait but negatively correlate with blood glucose levels and could thus be beneficially affecting glucose regulation [39].

Fecal microbiota analysis revealed that *Ruminococcaceae* became the predominant enterotype in dapagliflozin-treated rats, which is a family that produces SCFAs known to support metabolic health. Other functional predictions using both PICRUSt and KEGG indicated that the fecal bacteria in this group were more involved in glycerolipid metabolism, cytochrome P450 activity, and ion transport, suggesting microbial metabolism was shifting related to fat digestion and detoxification. As opposed to metformin, which did modulate their ratio and affected *Firmicutes*/*Bacteroidetes*, dapagliflozin has no consequence for those aspects but has demonstrated some changes in the fecal microbiome as a whole. Dapagliflozin exerts its modulation of fecal microbiota in a distinctly different way than metformin, which may provide an additional mechanism in support of its alleged antidiabetic effects, particularly in combination with metformin [39].

A study by Jin et al. indicates that dapagliflozin can address male fertility issues caused by diabetes via interactions with the gut microbiome and testicular metabolism. In particular, dapagliflozin relocated the bacterial colonies in the small intestine by inducing the growth of beneficial bacteria, including *Lactobacillus johnsonii*, and inhibiting potentially harmful bacteria, including *Oscillibacter* sp. and *Helicobacter rodentium*. The microbiological changes helped in mediating critical metabolic pathways, especially those involved in the adenosine signaling system, specifically the attempt to normalize the level of adenosine in the gut, cyclic AMP in blood, and 2′-deoxyinosine in the testes. The reduction in 2′-deoxyinosine is of particular importance because of its role in reducing cellular stress and mitigating sperm cell death and the ultimate loss of sperm. Sperm analysis under laboratory conditions indicated that dapagliflozin reduced the chance of sperm cells expressing proteins associated with cell death, increased the level of protective factors, and ultimately enhanced both the quality and quantity of sperm. Besides sperm analysis, there was also evidence collected from cell culture conditions that suggested 2′-deoxyinosine inhibited the beneficial effects of dapagliflozin and was associated with testicular injury. In summary, the results from this study suggested that dapagliflozin may have reproductive health benefits, which have previously been unaccounted for, as a pharmaceutical that provides benefits beyond controlling blood glucose level [40].

Dapagliflozin was found to be a dual-pronged approach for individuals living with diabetes by potentially addressing both a “metabolic problem” and reproduction through the gut–testis axis. Moreover, the drug’s balanced modulation of the gut microbiome and regulation of the adenosine metabolic pathway (specifically, adenosine/cyclic AMP/2′-deoxyinosine pathway) likely act as a vital component for normal spermatogenesis after protecting the testicular cells from damage. Even though the results from the mouse model are promising, there is substantial uncertainty about how and if they will translate into human patients. It will be important for future work to determine whether fertility benefits are distinct from the drug’s impact on diabetes control and whether similar outcomes could be achieved with microbiome-directed therapies, such as probiotic supplementation. This work also potentially opens the door for novel treatment combinations to address diabetes and its reproductive consequences simultaneously and improves knowledge regarding how gut health may provide changes influencing male fertility through metabolic regulation [40].

Moreover, dapagliflozin reduced myocardial ischemia–reperfusion injury (IRI) in diabetic rats using the gut microbiota–TMAO–ferroptosis mechanism. 16S rRNA sequencing showed that dapagliflozin altered gut microbiota composition by increasing *Bacteroidetes* (*Escherichia–Shigella* and *Prevotella*) while decreasing *Firmicutes*. Dapagliflozin increased abundances in gut microbiota known to correspond with reduced TMAO (gut metabolite that is associated with heart health). Additionally, dapagliflozin lowered the TMAO levels. Diabetic IRI rats had higher TMAO levels and higher markers for ferroptosis than control rats, and these outcomes were decreased by DAPA therapy. The authors used a network pharmacology approach and identified 11 genes (notably ALB, HMOX1, and PPARG) showing that DAPA therapy is associated with TMAO metabolism and ferroptosis regulation. Molecular docking results indicated TMAO had a strong interaction with DPP4 (docking score −5.44; MM-GBSA −22.02 kcal/mol), as noted in their findings, which could mean DAPA’s cardioprotection could have resulted from blocking an interaction between TMAO and DPP4. RT-PCR results showed DAPA treatment upregulated anti-ferroptosis genes (HMOX1 and PPARG) and downregulated proferroptosis genes (MAPK1 and DPP4), and functional enrichment analysis suggested pathways through AGE-RAGE and TNF signaling [41]. Dapagliflozin’s cardioprotective action in diabetes appears to be via a novel mechanism: gut microbiota modulation to reduce TMAO levels, thereby inhibiting ferroptosis in cardiomyocytes. Although these findings underscore dapagliflozin’s therapeutic promise for gut–heart axis modulation, limitations included a limited number of animals in the study (*n* = 9) and no validation in human volunteers [41].

### 5.2. Canagliflozin

The effects of canagliflozin on plasma uremic toxins and intestinal microbiota composition were investigated in a mouse model of CKD. The impaired renal function was not affected by two-week canagliflozin (10 mg/kg, p.o.) treatment; however, it significantly reduced the plasma levels of p-cresyl sulfate and indoxyl sulfate in renal failure mice (a 75% and 26% reduction, respectively). Moreover, canagliflozin significantly changed the microbiota composition in the renal-failure-induced mice. These results show that canagliflozin exerts intestinal effects that decrease the accumulation of uremic toxins, including p-cresyl sulfate, and the reduction of accumulated uremic toxins by canagliflozin could provide a therapeutic option in CKD [42].

Wang et al. studied the ability of canagliflozin to treat cardiovascular diseases (CVDs) in type 2 diabetic mice, as well as its mechanism of action. Mice with diabetic CVD were administered a high-fat diet for 24 weeks, followed by oral gavage with metformin (200 mg/kg/day) or canagliflozin (50 mg/kg/day) for 6 weeks. Serum lipid accumulation decreased following canagliflozin and the arteriosclerosis index and atherogenic index of plasma were reduced. Additionally, canagliflozin treatment hindered the circulating markers of inflammation. Interestingly, cardiac mitochondrial homeostasis was found to be enhanced by canagliflozin. Moreover, canagliflozin treatment relieved oxidative stress in diabetic CVD mice. Regarding the gut microbial composition, canagliflozin augmented the ratio of *Firmicutes*/*Bacteroidetes* and the relative abundance of *Alistipes*, *Olsenella*, and *Alloprevotella*, while it decreased the abundance of *Mucispirillum*, *Helicobacter*, and *Proteobacteria* at various taxonomic levels in mice with diabetic CVD. Collectively, canagliflozin treatment changed the colonic microbiota composition almost to the normal level, which was linked to blood lipids, inflammation, and oxidative stress and might play an important role in CVD [34].

Furthermore, canagliflozin administered at a dose of 20 mg/kg affects gut microbiota and salt-sensitive-hypertension-induced kidney injury in Dahl salt-sensitive (DSS) rats. Rats were fed with a high-salt diet to induce hypertension and kidney injury, and physical and physiological indicators were then assessed. In addition, 16S rRNA sequencing and liquid chromatography–tandem mass spectrometry (LC–MS/MS)-based metabolic profiling integrated with advanced differential and association analyses were performed to investigate the link between the microbiome and the metabolome in male DSS rats. A high-salt diet altered the balance of the intestinal flora and increased toxic metabolites (methyhistidines, creatinine, homocitrulline, and indoxyl sulfate), leading to severe kidney damage. Canagliflozin contributed to enhancing the intestinal flora of DSS rats by significantly enhancing cardiac mitochondrial homeostasis by increasing the amount of *Corynebacterium* spp., *Bifidobacterium* spp., *Facklamia* spp., *Lactobacillus* spp., *Ruminococcus* spp., *Blautia* spp., *Coprococcus* spp., and *Allobaculum* spp. Moreover, the reconstruction of the intestinal microbiota led to critical changes in host amino acid metabolite concentrations. The concentration of uremic toxins, such as methyhistidines, creatinine, and homocitrulline, in the serum of rats was reduced by canagliflozin, which resulted in oxidative stress and renal injury alleviation [43].

Diabetic mice in a study by Zeng et al. received canagliflozin by gavage for 8 weeks. Collection of feces was performed for 16s rRNA gene sequencing and LC-MS/MS analysis and enriched metabolic pathways through Kyoto Encyclopedia of Genes and Genomes (KEGG). Liver, muscle, intestinal, and fat were collected for qRT-PCR according to KEGG-enriched metabolic pathways. The results demonstrate that canagliflozin increased GLP-1 levels and impacted the composition of gut microbiota and metabolites. It mainly increased *Muribaculum*, *Ruminococcaceae*_UCG-014, and *Lachnospiraceae*_UCG-001, decreased ursodeoxycholic acids and hyodeoxycholic acids, and increased fatty acid metabolites in feces. Analysis of the changes of intestinal microbial composition and metabolites in diabetic mice after canagliflozin intervention revealed that canagliflozin affected intestinal fatty acid and bile acid metabolism [44].

### 5.3. Empagliflozin

Neuroinflammation is a process which occurs in the CNS and can be caused by several factors such as a high-fat diet. This causes alteration in gut microbiota and increases the permeability of the intestine. The antidiabetic drug empagliflozin is used traditionally to regulate glucose level, but it has been observed that it can modulate microbiota, suppress the levels of certain bacterial groups, and reduce neuroinflammation. Furthermore, in mice fed a high-fat diet, empagliflozin restored the genes that had been damaged. According to the data, it appears that the drug may help reduce CNS inflammation by modifying the gut microbiome, inhibiting the stimulation of specific glial cells, and decreasing the secretion of proinflammatory factors. The protective mechanism of the drug could be associated with its capacity to initiate cleanup processes in the cells through the Akt-mTOR signaling pathway [45]. Moreover, obesity can result in a variety of health problems, including obesity-related kidney difficulties. Empagliflozin effectively decreases body fat, and this helps in decreasing the incidence of obesity-related glomerulopathy. Findings indicated that the drug exhibits kidney-protective effects, and the mechanism is linked to lipid metabolism. The suppression of glycerol 3-phosphate and CoA production is heavily dependent on empagliflozin’s alteration of the gut microbiota. Empagliflozin use made significant improvements by lowering glomerular size and renal fat buildup, which returned renal morphology to normal. Empagliflozin-treated mice displayed a higher abundance of specific gut bacteria and a decline in other microbial populations. This suggests that the gut microbiota could be altered as a result of empagliflozin treatment. These findings may imply that the gut–kidney axis may be regulated by empagliflozin, providing renoprotective effects [46].

A study by Guan et al. examined how gut bacteria and their metabolites contribute to changes in gut microbiota in patients on empagliflozin who do not have a decreased ejection fraction. The clinical trial assessed the extent of blood SCFAs, gut microbiota which are SCFA-producing, and sST2, a marker of fibrosis, to determine whether SGLT-2 is capable of reducing cardiac fibrosis by gut microbiota. A hypothetical SGLT-2–gut–HF axis may be determined from the above information [47]. The most common and dangerous chronic condition is diabetes and raises the risk of cognitive impairment. It has been shown that gut bacteria are important in the progression of diabetes and cognitive dysfunction. The impact of empagliflozin on gut microbiota and cognitive decline in diabetic mice was examined. Empagliflozin improved the cognitive impairment of mice as evidenced by behavioral tests. Empagliflozin also restored gut bacteria structure in diabetic mice according to 16S rRNA gene sequencing. In diabetic mice, the diversity and makeup of the gut microbiota were markedly altered by empagliflozin treatment. Empagliflozin treatment showed a lower number of harmful bacteria (like *Helicobacter*) and a significantly higher number of beneficial bacteria (like *Lactobacillus*). Thus, empagliflozin may have an impact on the gut microbiota in diabetic mice, which may then have an impact on cognitive function through mechanistic pathways in the gut microbiota [48].

### 5.4. Sotagliflozin

**Figure 2:**
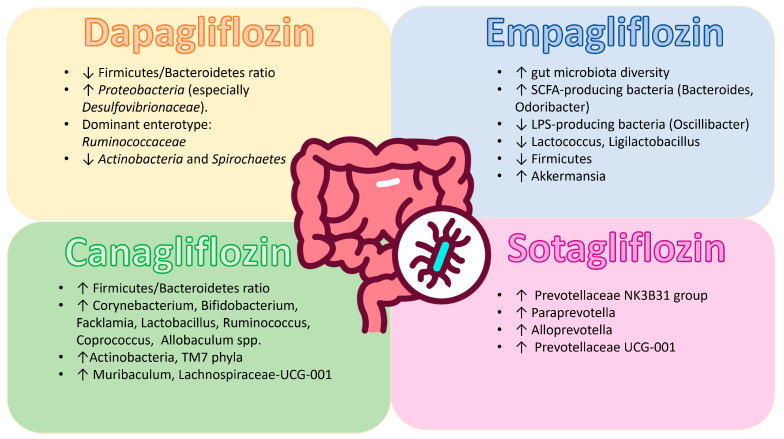
The effect of SGLT-2 inhibitors on gut microbiota species.

Myocardial infarction (MI) and depression are highly co-morbid and exacerbate health risk when occurring together. In their study, Liao et al. explored whether the dual SGLT-1/2 inhibitor sotagliflozin would augment cardiac function and depression-like behaviors after MI. In a mouse model, the investigators found that sotagliflozin significantly improved cardiac function and reduced depressive behaviors. Most alterations were increases in certain bacterial groups like *Alloprevotella*, *Prevotellaceae* UCG-001, and *Prevotellaceae* NK3B31, which were linked to changes in the gut microbiota. On the other side, sotagliflozin did not significantly change alpha diversity but did induce clear changes in microbial composition (beta diversity). As a means to assess whether or not the gut microbiota was directly implicated in these effects, FMT was carried out. Mice that received sotagliflozin exhibited heart improvement in alignment with microbial composition change. All of these results suggest that the gut microbiota is most likely to be the main mechanism of sotagliflozin’s cardiac-protective effects. Furthermore, the advantages may have originated from certain bacterial species that were shown to have specific metabolic roles, such as the synthesis of SCFA and the metabolism of thyroid hormones, as predicted by functional property predictions [49] (Figure 2).

## 6. Conclusions

SGLT-2 inhibitors have emerged not only as effective antihyperglycemic agents but also as modulators of cardiovascular and renal outcomes in type 2 diabetes mellitus. Growing evidence further suggests that their therapeutic benefits may extend to the regulation of gut microbiota, a central player in metabolic, immune, and inflammatory processes. The interplay between SGLT-2 inhibitors and gut microbial communities could represent an additional mechanism contributing to their systemic effects. Collectively, the current literature underscores the potential of SGLT-2 inhibitors to influence host physiology beyond glucose homeostasis through microbiome-mediated pathways.

## 7. Knowledge Gaps and Future Directions

Despite these promising insights, several important gaps remain. First, most available evidence is derived from preclinical models or small-scale clinical studies, limiting the generalizability of findings. Second, the causal mechanisms by which SGLT-2 inhibitors shape gut microbiota composition and function are not fully elucidated. It remains unclear whether these changes are direct effects of the drugs, secondary to altered glucose handling, or mediated by other systemic factors. Third, inter-individual variability in microbiome response, influenced by diet, genetics, and co-morbidities, has not been systematically explored. Finally, the clinical relevance of microbiota modulation in mediating the cardiovascular, renal, and metabolic benefits of SGLT-2 inhibitors remains to be confirmed in large, longitudinal human studies. Future research should prioritize well-designed clinical trials integrating multiomics approaches (metagenomics, metabolomics, transcriptomics) to unravel causal pathways. Investigating patient-specific microbial signatures predictive of therapeutic response may open the door to personalized, microbiota-targeted strategies. Additionally, SGLT-2 inhibitors need to be tested for their efficacy in hematopoietic disorders. Ultimately, clarifying the SGLT-2 inhibitor–gut microbiome axis may not only enhance understanding of their pleiotropic benefits but also pave the way for novel microbiome-based interventions in cardiometabolic disease management.

## Figures and Tables

**Table 1:** The effect of SGLT-2 inhibitors on gut microbiome and their effect on different body organs.

Drug	Dosing	Species	Main Findings	Reference
Dapagliflozin	1 mg/kg/day, i.p., 8 weeks	Mice	↓ Cardiac fibrosis. ↑ Endothelial function. ↓ *Firmicutes*/*Bacteroidetes* ratio.	[38]
Dapagliflozin	1 mg/kg/day, oral, 4 weeks	Rats	Enriched *Proteobacteria* (especially *Desulfovibrionaceae*); no increase in beneficial bacteria (*Lactobacillaceae, Bifidobacteriaceae*). Dominant enterotype: *Ruminococcaceae*; reduced *Actinobacteria* and *Spirochaetes*. ↑ Glucose tolerance (↓ fasting/postprandial glucose, ↓ HOMA-IR)	[39]
Dapagliflozin	1 mg/kg, oral, 5 weeks	Mice	↑ Sperm quality (concentration/motility). ↓ Apoptosis/oxidative stress. Modulation of gut microbiota–testis axis.	[40]
Dapagliflozin	30 μM (in vitro)	GC-2 cells	↓ Palmitic-acid-induced apoptosis. ↓ ROS. Effects were reversed by 2′-deoxyinosine.	[40]
Dapagliflozin	40 mg/kg/day, i.p., 7 days	Rats	↓ TMAO levels in heart tissue. Modulation of gut microbiota (↑ *Bacteroidetes*, ↓ *Firmicutes*). Regulation of ferroptosis-related genes (↑ ALB, HMOX1, PPARG, CBS, LCN2, PPARA; ↓ MAPK1, MAPK8, PARP1, SRC, DPP4). Molecular docking showed strong binding between TMAO and DPP4 (docking score: −5.44).	[41]
Canagliflozin	10 mg/kg, oral, 2 weeks	Mice	↓ Plasma uremic toxins (PCS, IS). ↑ Cecal SCFAs. Modulation of gut microbiota (↓ *Bifidobacterium*, ↑ *Actinobacteria*, TM7 phyla).	[42]
Canagliflozin	50 mg/kg/day, oral gavage, 6 weeks	Mice (with diabetic CVD induced by high-fat diet)	↓ Serum lipid accumulation. ↓ Circulating inflammation markers. ↑ Cardiac mitochondrial homeostasis. ↓ Oxidative stress. ↓ Myocardial injury. Modulation of colonic microbiota composition (↑ *Firmicutes*/*Bacteroidetes* ratio).	[34]
Canagliflozin	20 mg/kg/d, oral, 12 weeks	Dahl salt-sensitive (DSS) rats	↓ Salt-sensitive hypertension. ↓ Renal damage. ↓ Oxidative stress. Modulation of intestinal flora (↑ *Corynebacterium*, ↑ *Bifidobacterium*, ↑ *Facklamia*, ↑ *Lactobacillus*, ↑ *Ruminococcus*, ↑ *Blautia*, ↑ *Coprococcus*, ↑ *Allobaculum* spp.). ↓ Uremic toxins (methyhistidines, creatinine, homocitrulline, indoxyl sulfate).	[43]
Canagliflozin	10 mg/kg/d, oral gavage, 8 weeks	db/db mice (type 2 diabetic mice)	↑ GLP-1 level. Modulation of gut microbiota (↑ *Muribaculum*, ↑ *Ruminococcaceae*_UCG-014, ↑ *Lachnospiraceae*_UCG-001). Influence on intestinal fatty acid and bile acid metabolism. ↓ UDCA and HDCA. ↑ Fatty acid metabolites in feces.	[44]
Empagliflozin	2 mg/kg (low) or 6 mg/kg (high), daily for 4 weeks	Mice	↓ Neuroinflammation and astrocyte activation in high-fat diet (HFD) mice. ↑ Gut microbiota composition (↓ *Lactococcus*, *Ligilactobacillus*). ↑ Synaptophysin expression.	[45]
Empagliflozin	10 mg/kg, oral, 8 weeks	Mice	↑ Kidney function and reduced lipid accumulation in obesity-related glomerulopathy (ORG). Modulation of gut microbiota (↓ *Firmicutes*, increased *Akkermansia*). Regulated lipid metabolism pathways (glycerophospholipid, CoA biosynthesis).	[46]
Empagliflozin	10 mg/day orally for 6 months	Human patients with HFpEF	↑ Gut microbiota diversity and SCFA levels. ↓ Inflammation and myocardial fibrosis in HFpEF.	[47]
Empagliflozin	10 mg/kg/day, oral, 4 weeks	Mice	↓ Blood glucose and UACR. Restored gut microbiota diversity ↑ SCFA-producing bacteria (*Bacteroides*, *Odoribacter*) ↓ LPS-producing bacteria (*Oscillibacter*). ↑ Intestinal barrier function (↑ ZO-1, ↑ Occludin).	[48]
Sotagliflozin	30 mg/kg/day orally, 7 days before and 25 days after MI surgery	Mice	↓ Cardiac dysfunction. ↓ Depression-like behaviors (TST and FST). ↓ Infarct size and fibrosis Gut microbiota modulation: ↑ Alloprevotella, Prevotellaceae UCG-001, NK3B31 group	[49]

Downward arrows mean decrease and upward arrows mean increase.

## Data Availability

No new data were created or analyzed in this study.
